# Modeling and experimental insight into the electronic and structural properties of Sodium alginate/Polypyrrole/Titanium dioxide nanocomposites

**DOI:** 10.1038/s41598-026-53676-0

**Published:** 2026-05-28

**Authors:** Amira M. Salem, Reem A. Hassan, Nourhan M. S. Abd El-Rahman, Hanan Elhaes, Medhat A. Ibrahim

**Affiliations:** 1 Higher Institute of Optics Technology HIOT, Sheraton Heliopolis, Cairo, 11799 Egypt; 2 Physics Department, Faculty of Science, Ain Shams University, Cairo, 11757 Egypt; 3 Chemistry Department, Faculty of Science, Cairo University, Giza, 12613 Egypt; 4 Physics Department, Faculty of Women for Arts, Science and Education, Ain Shams University, 11757 Cairo, Egypt; 5 Spectroscopy Department, National Research Centre, 33 El-Bohouth St., 12622, Dokki, Giza, Egypt; 6 Center for Converging Sciences and Emerging Technologies (CoSET), Benha National University (BNU), El-Obour, 13518 Egypt; 7 Molecular Modeling and Spectroscopy Laboratory, Centre of Excellence for Advanced Science, National Research Centre, 33 El-Bohouth St., 12622, Dokki, Giza, Egypt

**Keywords:** DFT, Sodium alginate, Conductive polymers and TiO_2_, Chemistry, Materials science

## Abstract

The creation of functional materials which enable adjustable electronic properties was fundamental to the development of electronic sensors and biomedical applications. The research assesses a ternary nanocomposite system which combines sodium alginate (SA) with polypyrrole (PPy) and titanium dioxide (TiO_2_) through dual methods of computational simulation and experimental testing. The researchers used Density Functional Theory (DFT) simulations at the B3LYP/6-31G(d, p) level to study how molecules interact with each other and how their electronic structures behave. The research demonstrates that the SA/PPy/TiO_2_ composite material exhibits better electronic performance because it shows both a smaller energy gap and a smaller total dipole moment. The global reactivity indices which include ionization energy, chemical hardness, and the HOMO-LUMO gap reveal a synergistic effect which enhances charge transfer according to Density of States (DOS) measurements and Quantum Theory of Atoms in Molecules (QTAIM) results. The researchers used FTIR and UV-Vis spectroscopy to confirm that SA/TiO_2_ composite films matched theoretical predictions at a high accuracy. The B3LYP/6-31G(d, p) level shows that it successfully detects a smaller HOMO–LUMO gap which demonstrates that the studied composites exhibit greater chemical reactivity and better internal charge transfer and higher electrical conductivity.

## Introduction

The need for multifunctional materials that have eco-friendly characteristics has increased during recent years because of technological advancements, global sustainability objectives, and environmental conservation efforts^1^. The natural polymer Sodium Alginate (SA) which commercial enterprises extract from brown algae^2^ serves as the main material that meets these requirements because it possesses non-toxic properties and biocompatibility together with its ability to break down in nature^3^. Engineers develop SA-based materials into different designs which serve vital functions in water treatment processes and energy storage systems^4^. Through the creation of SA composites as renewable resources engineers can develop environmentally friendly solutions which help reduce the need for nonrenewable materials and decrease the ecological damage caused by conventional resource-intensive products^5^. Researchers investigate new transparent conducting materials to meet the rising demand for high-performance electronic products. The combination of SA with conductive polymers^6^ and metallic grids^7^ and various nanofillers^8^ enables researchers to design materials which maintain high electrical conductivity together with visibility in both the visible and infrared light ranges^9^. Polypyrrole (PPy) operates as an excellent conductive polymer because of its outstanding electrical conductivity together with its ability to store large amounts of energy and its simple production process^10–13^. PPy finds various applications in chemical sensors and electronic devices^14^ yet its practical usage gets restricted by its weak mechanical performance and inadequate thermal resistance and the formation of mechanical instability which occurs after the doping process^15^. The material needs stabilizing matrices to enable its use beyond its purpose because of its fixed structure and non-biodegradable properties^16^. The non-biodegradable and rigid structure of Titanium dioxide (TiO_2_) together with its high performance as a semiconductor makes it one of the most researched semiconductor materials in existence^17,18^. TiO_2_ functions effectively in harsh conditions because of its strong chemical and thermal stability which enables its performance in extreme environments^19^. Nano-sized TiO_2_ provides multiple benefits because it has a large specific surface area and strong adsorption capacity together with excellent properties for dispersion^20^. Researchers explore different modification techniques to overcome the limitations imposed by its broad band gap which restricts its real world applications^21^. Scientists have investigated how to use conductive polymers to change the properties of TiO_2_ through their research work. The visible light absorption and charge transfer enhancement which PPy provides lead to a decrease in band gap energy for PPy/TiO_2_ nanocomposites compared to pure TiO_2_^22^. The introduction of TiO_2_ in a PPy matrix results in enhanced electronic characteristics for the resulting nanocomposite material^23^. The system uses SA as a biopolymer matrix to create better stability for the PPy/TiO_2_ system because it maintains consistent material distribution and improves environmental friendliness^24^. The combination of these materials has been used to create new sensors and supercapacitors through the combination of PPy/TiO_2_/ZnO composites^25^. Density Functional Theory (DFT) serves as an effective tool for studying the electronic structures of complex molecular systems^26,27^. DFT offers polymer reactivity indicators which help researchers understand how polymers function according to the findings from experimental research^28,29^. The total dipole moment and HOMO/LUMO energy gaps and electrostatic potential and density of states (DOS) results from DFT calculations function as essential descriptors which define the primary surface and molecular attributes of a chemical structure^30–35^. The reactivity of these structures together with their stability can be evaluated through HOMO/LUMO indicators which combine with Quantum Theory of Atoms in Molecules (QTAIM) analysis to provide an in-depth assessment^36^. The previous computational studies demonstrated that DFT could model multiple interactions which included both the changes in poly(lactic acid) structures and the detection of Cl_2_ gas using MgO nanotubes^37,38^. The Pure2 DopeNet neural network enables users to predict the physical properties of doped materials with quantum mechanical precision because it uses modern computational methods^39^. DFT has been used as a fundamental tool for analyzing how triatomic gases, including NO_2_, SO_2_, and CO_2_, behave when they adsorb onto surfaces^40,41^. DFT and molecular docking techniques have been applied in biomedical research to determine whether nanocomplexes and nanocages, such as B_12_N_12_, can be used as drug carriers for Allicin^42^ and Hydroxytyrosol^43^.

This study aims to comprehensively investigate how the SA/PPy/TiO₂ ternary composite performs through its complete testing process because the study wants to find out how the composite functions through its electronic connections which exist between two different materials. The DFT: B3LYP/6-31G(d, p) was used together with experimental characterization to create a precise mapping of molecular interactions and essential electronic properties which include kinetic stability and charge transfer efficiency. The research introduces a method which uses predictive modeling through a computational framework that scientists used to benchmark against experimental FTIR and UV-Vis spectra of SA/TiO₂ films for ternary system prediction verification. The study creates fundamental knowledge about polymer-semiconductor interfaces through its demonstration that PPy addition leads to smaller HOMO–LUMO gaps. The work presents a method for developing advanced materials which will be useful in high-sensitivity sensing technology and optoelectronic devices and biomedical research.

## Materials and methods

### Calculation details

The optimization of the model molecules representing SA, PPy, TiO_2_ and their composites were conducted using the Gaussian 09 (G09) software at the Molecular Modeling and Spectroscopy Laboratory, National Research Centre (NRC), Egypt^44^. DFT was employed for this optimization, utilizing the B3LYP a hybrid exchange–correlation functional^45–47^ with the 6-31G (d, p) basis set. This combination was selected for its favorable balance of accuracy and computational efficiency, widely recognized for its effectiveness in predicting molecular geometries and electronic characteristics. The B3LYP/6-31G(d, p) level is a standard benchmark in literature for studying the electronic structures of complex molecular systems^48,49^.

Following optimization, DFT/B3LYP methodology was used to calculate critical molecular properties, such as the total dipole moment (TDM), the molecular electrostatic potential (MESP), and the HOMO/LUMO energy gap ΔE. These calculations were made using the 6-31G (d, p) basis set and are essential for evaluating the molecules’ reactivity and interaction potentials. The DFT/B3LYP method is generally reliable and although it may not be able to effectively describe weak interactions because they are by nature more complex; nonetheless, it remains a prominent choice in the literature for its capability to provide valuable insights into molecular behavior. TD-SCF and DFT/B3LYP/6-31G(d, p) were used to compute the UV-Vis spectra for SA and SA/TiO_2_.

### Chemicals and reagents

SA was acquired from Spain’s PANAREAC QUIMICA Barcelona. Copper chloride and zinc acetate were obtained from Sigma-Aldrich Company, Inc. in the United States. Ethanol, acetic acid (CH_3_COOH), sodium hydroxide, polyethylene glycol (M.W 6000), and ethyl alcohol (C_2_H_5_OH) were purchased from El Nasr Pharmaceutical Chemicals Co. in Cairo, Egypt. TTIP, or titanium tetraisopropoxide Ti (OC_3_H_7_)_4_, was acquired from Sigma Aldrich in Germany. Graphite powder, potassium permanganate (99%), phosphoric acid (85%), sodium hydroxide (≥ 97%), urea (97%) and citric acid (99.5%) were sourced from Fisher Chemical. In the meantime, PIOCHEM provided 30% hydrogen peroxide and Scharlau provided 96% sulfuric acid. In this experiment, deionized (DI) Milli-Q water and distilled water were employed.

### Synthesis of Titanium dioxide nanoparticles

The precipitation method was used to create nano TiO_2_. In an ice bath, titanium tetraisopropoxide was dissolved in 100% ethanol. Water was then added to the mixture while it was being stirred slowly. To limit the hydrolysis process and, in turn, regulate the grain growth, acetic acid and polyethylene glycol were added to the solution drop by drop. The solution was agitated vigorously for two hours and overnight to facilitate precipitation while the acetic acid was added. Following that, a two-layer solution was created, with the organic byproduct of the hydrolysis in the top layer and a TiO_2_ precipitate in the bottom. The precipitate was filtered, repeatedly cleaned with distilled water, and then drying overnight at 100 °C. To obtain a pure anatase TiO_2_ nanoparticle, the obtained yellow block crystals were crushed, ground into a fine powder, and then calcined for three hours at 500 °C.

### Synthesis of SA/TiO_2_ nanoparticles

At 50 °C, 0.5 g of SA powder was dissolved in 25 mL of double-distilled water while being stirred for 30 min to produce a viscous solution that was suitably clean. Following the dissolution of SA, TiO_2_ was added and continuously stirred under the same conditions. Different concentrations of TiO_2_ (0, 0.5, 1, 1.5, 2, and 5 wt%) were then added to the SA solution; these were labeled as Pure SA, SA/0.5 wt% TiO_2_, SA/1 wt% TiO_2_, SA/1.5 wt% TiO_2_, SA/2 wt% TiO_2_ and SA/5 wt% TiO_2_ respectively. The samples were put on clean glass Petri plates and allowed to dry in the air for six days to remove any remaining solvent and create a pure SA film for comparison.

### Characterization techniques

Using a Vertex 80 FT-IR spectrometer (Bruker Optics GmbH, Germany), Fourier transform infrared spectroscopy (FTIR) spectra were collected at room temperature for the Pure SA, SA/0.5 wt% TiO_2_, SA/1 wt% TiO_2_, SA/1.5 wt% TiO_2_, SA/2 wt% TiO_2_ and SA/5 wt% TiO_2_ films respectively. In the spectral region of 4000–400 cm^− 1^, it was fitted with a diamond ATR crystal system to study their structures.

The UV–Vis–NIR diffuse reflectance spectra of the samples were measured at ambient temperature using a Jasco V-570 spectrophotometer, covering the wavelength range of 190 to 2500 nm.

## Results and discussions

### Starting model molecules

**Fig. 1:**
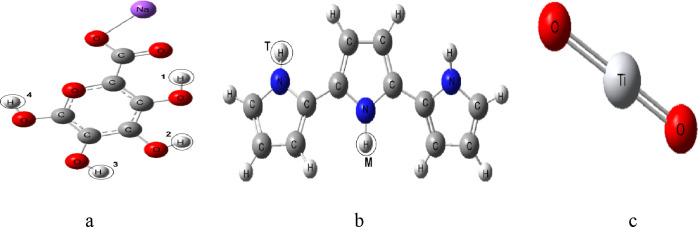
Proposed model structures whereas (**a**) Sodium alginate SA, (**b**) Polypyrrole PPy consists of 3 units and (**c**) Titanium dioxide TiO_2_.

As depicted in Fig. 1, three proposed structures were constructed as the starting and/or building model molecules. One molecule was dedicated to SA (Fig. 1: a), consisting of one repeat unit, while another represented PPy (Fig. 1: b), composed of 3 repeat units. Additionally, a molecule for TiO_2_ (Fig. 1: c) was created. Several physical parameters were calculated, including the Total dipole moment (TDM), the HOMO/LUMO energy gap (ΔE), as well as the Molecular Electrostatic Potential (MESP) maps for each optimized structure.

### Computed physical parameters

The calculated physical parameters encompass TDM, ΔE, and MESP maps. These physical parameters are crucial for evaluating the reactivity and stability of the SA-based composites.

**Table 1:** Calculated total dipole moment TDM in (Debye), HOMO/LUMO energy gap ΔE in (eV) for the studied SA, PPy and TiO_2_ structures.

Structure	TDM (Debye)	ΔE (eV)
SA	7.8564	1.4147
PPy	1.4279	4.3815
TiO_2_	6.6237	3.4956

TDM determines a molecule’s polarity and potential for interactions with other molecules by measuring the separation of positive and negative charges within it. As shown in Table 1, the calculated TDMs for pure SA reveal a TDM of 7.8564 Debye, while PPy shows a lower value of 1.4279 Debye. TiO_2_ has a TDM of 6.6237 Debye.

The HOMO/LUMO energy gap serves as a measure of a material’s electronic properties and its reactivity. SA has a relatively low band gap of 1.4147 eV, as reported experimentally indirect energy gap of SA^50^, Indicating a higher potential for electron transfer and thus greater chemical reactivity. In contrast, PPy presents a much larger band gap of 4.3815 eV, suggesting lower reactivity. TiO_2_ has a band gap of 3.4956 eV as in the range reported of TiO_2_ as reported in^51^.

**Fig. 2:**
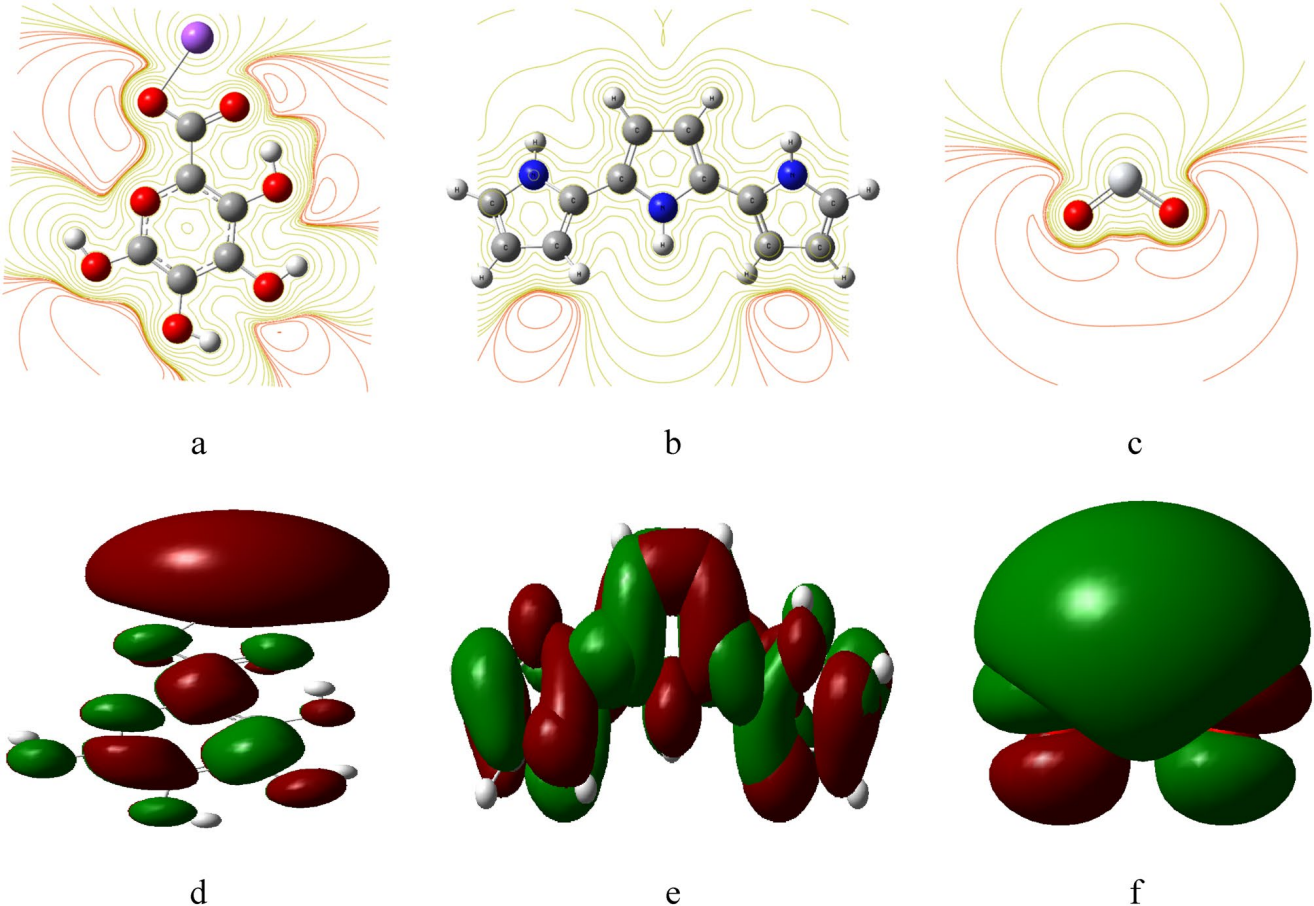
Calculated MESP maps for (**a**) SA, (**b**) PPy consists of 3 units, (**c**) TiO_2_ Calculated HOMO/LUMO energy gap for the modeled structure for: (**d**) SA, (**e**) PPy consists of 3 units and (**f**) TiO_2_.

MESP maps provide an insight into the electronic behavior of the analyzed model molecules. Each configuration illustrates how molecular arrangements affect the distribution of electrostatic potential, which was vital for predicting reactivity and stability.

In Fig. 2 (a-c), the MESP maps highlight differences between pure materials like SA, PPy, and TiO₂. For example, SA shows a distinct charge distribution that may enhance its reactivity in certain environments due to its negative regions. PPy, with its higher band gap, indicates a more stable structure with less tendency for electron transfer compared to SA, as evidenced by its MESP map. These MESP maps indicate the electronic properties of individual model structure and highlight the significance of their interactions in influencing the overall chemical behavior of the materials. This study can help scientific researchers create and improve materials for certain uses, like electronic devices, sensors, and catalysts. The calculated HOMO/LUMO band gap energies presented in Fig. 2 (d-f) can indicate the electron transfer capability of each structure. For instance, SA shows a certain electronic behavior that may enhance its reactivity, while PPy, particularly in its various configurations with SA, reveals how the interactions can modify electronic properties.

### Building SA/PPy model molecules

**Fig. 3:**
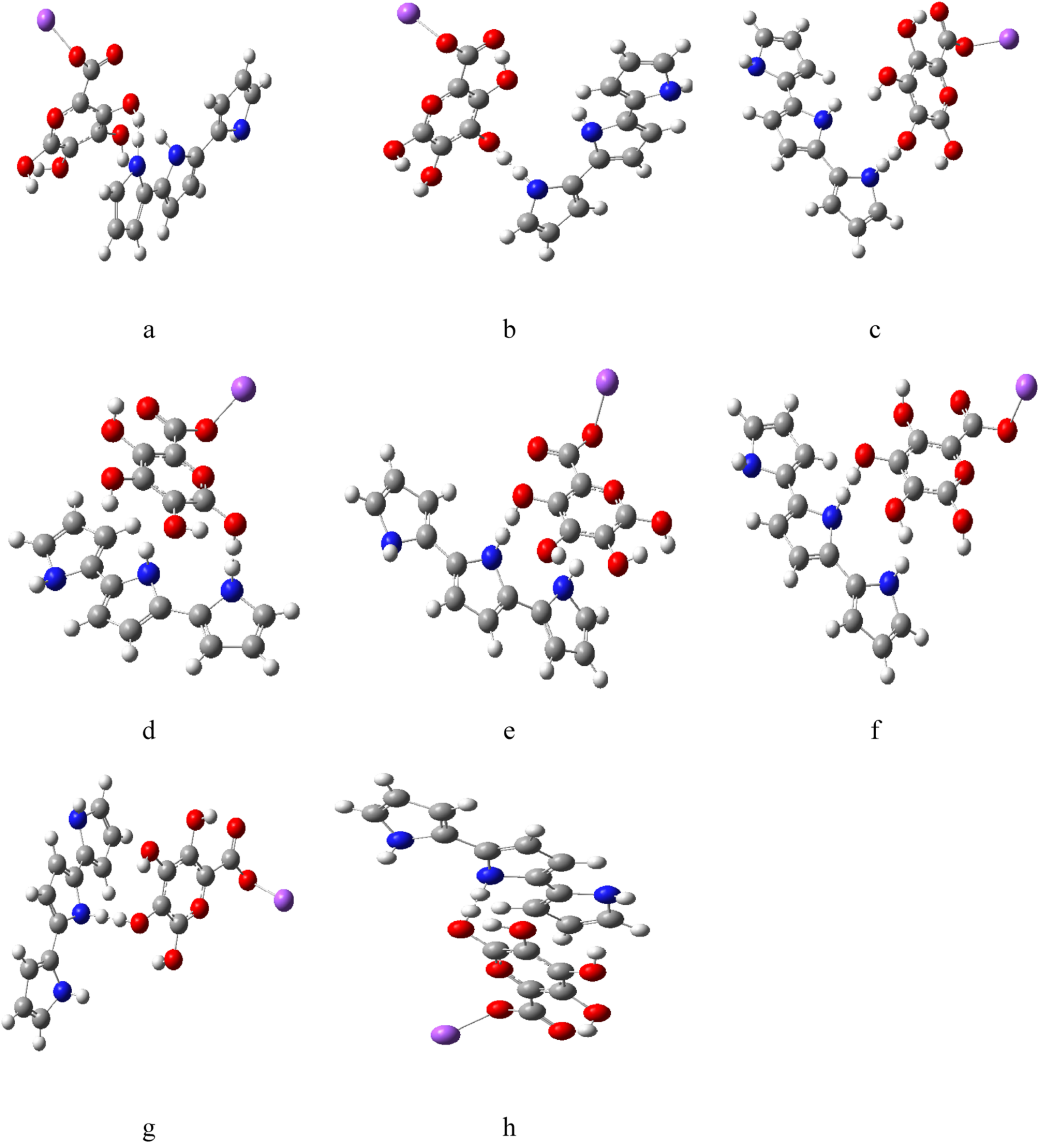
Proposed structures: (**a**) SA (H1) with PPy (T), (**b**) SA (H2) with PPy (T), (**c**) SA (H3) with PPy (T), (**d**) SA (H4) with PPy (T), (**e**) SA (H1) with PPy (M), (**f**) SA (H2) with PPy (M), (**g**) SA (H3) with PPy (M), (**h**) SA (H4) with PPy (M).

The model molecules namely Sodium alginate (SA) and the Sodium alginate/Zinc oxide (ZnO) composites were built with GausView 5.0 software^52^. All the calculated model structures were visualized also with GausView 5.0 software^52^. As illustrated in Fig. 1, the four hydrogen atoms on SA (Fig. 1: a) are numbered (H1, H2, H3 and H4) to indicate their positions. It was also crucial to specify whether the nitrogen atoms in PPy (Fig. 1: b) are located at terminal (T) or middle (M) positions, as this distinction significantly influences the potential bonding interactions with the hydrogen atoms. To explore the interactions between SA and PPy, eight building molecules were generated as shown in Fig. 3. The eight proposed structures are named a- SA (H1) with PPy (T), b- SA (H2) with PPy (T), c- SA (H3) with PPy (T), d- SA (H4) with PPy (T), e- SA (H1) with PPy (M), f- SA (H2) with PPy (M), g- SA (H3) with PPy (M), h- SA (H4) with PPy (M) respectively.

Notably, the combinations of SA with PPy in different configurations yield a range of TDMs, with SA (H3) with PPy (T) reaching the highest value of 10.0113 Debye as shown in Table 2. The various combinations of SA with PPy show a range of band gap energies, the gap of SA (H1) with PPy (T) was 1.7072 eV, which remains low and indicative of favorable electron transfer.

**Table 2:** Calculated TDM in (Debye), ΔE in (eV) for the studied SA/PPy composite structures.

Structure	TDM (Debye)	ΔE (eV)
SA (H1) with PPy (T)	9.4631	1.7072
SA (H2) with PPy (T)	9.7044	1.7404
SA (H3) with PPy (T)	10.0113	1.8778
SA (H4) with PPy (T)	6.2203	2.3363
SA (H1) with PPy (M)	5.9068	1.6705
SA (H2) with PPy (M)	6.2225	2.3352
SA (H3) with PPy (M)	8.4078	1.8041
SA (H4) with PPy (M)	6.2198	2.3363

**Fig. 4:**
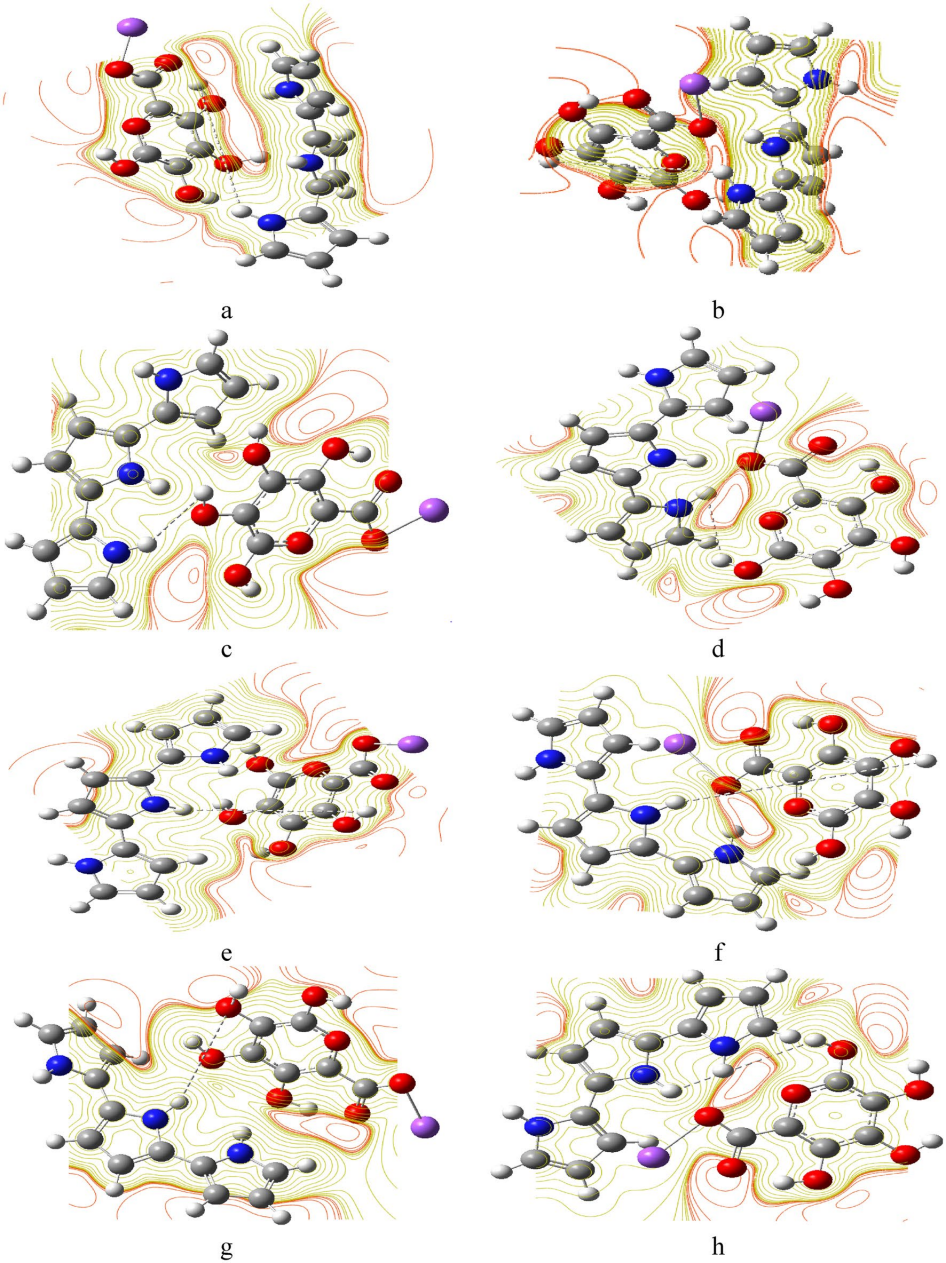
MESP for (**a**) SA (H1) with PPy (T), (**b**) SA (H2) with PPy (T), (**c**) SA (H3) with PPy (T), (**d**) SA (H4) with PPy (T), (**e**) SA (H1) with PPy (M), (**f**) SA (H2) with PPy (M), (**g**) SA (H3) with PPy (M), (**h**) SA (H4) with PPy (M).

**Fig. 5:**
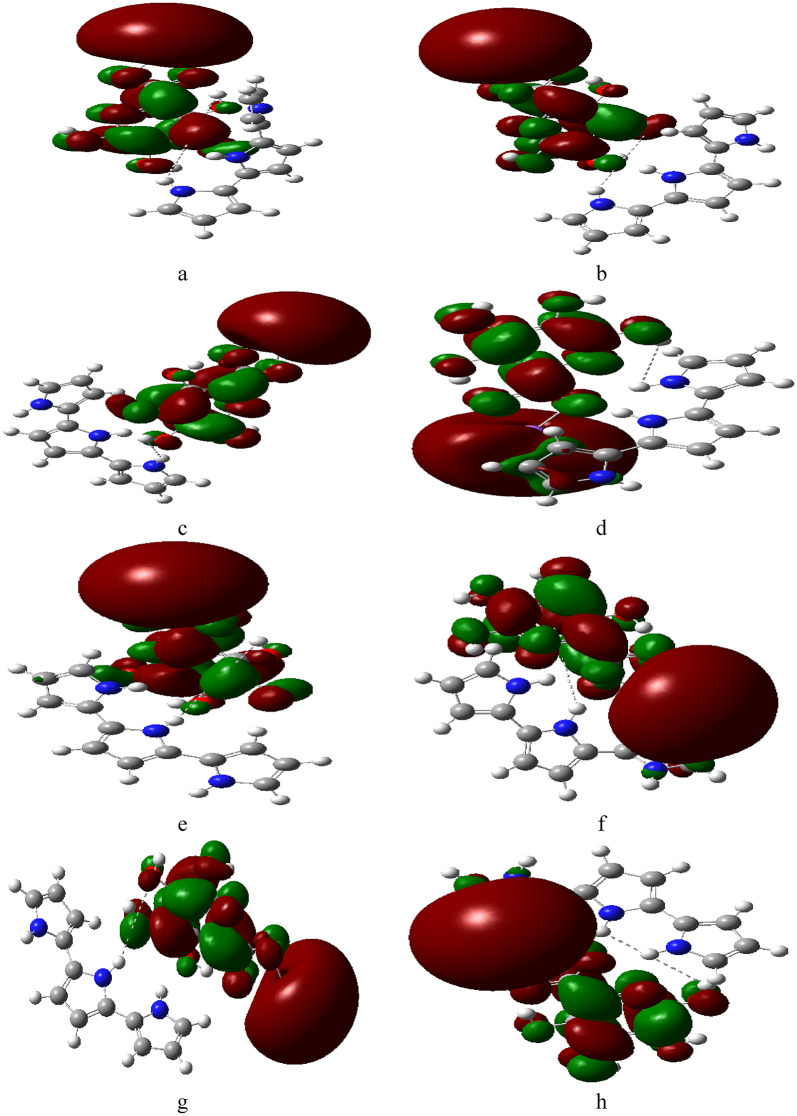
Calculated HOMO/LUMO energy gap for (**a**) SA (H1) with PPy (T), (**b**) SA (H2) with PPy (T), (**c**) SA (H3) with PPy (T), (**d**) SA (H4) with PPy (T), (**e**) SA (H1) with PPy (M), (**f**) SA (H2) with PPy (M), (**g**) SA (H3) with PPy (M), (**h**) SA (H4) with PPy (M).

Figure 4 depicts the combinations of PPy with SA and reveals how interactions create new electrostatic landscapes, potentially lowering energy barriers for chemical reactions. The maps also provide insights into the stability of different configurations, with areas of significant positive potential being favorable for bonding interactions. The maps also provide insights into the stability of different configurations. For instance, configurations like SA (H1) with PPy (T) (Fig. 4: a) showcase areas of significant positive potential, which could be favorable for bonding interactions. The combinations of SA with PPy, as illustrated in Fig. 5, display varying HOMO/LUMO band gap energies. These variations suggest that the integration of SA with PPy can lead to materials with tailored electronic characteristics, which could be beneficial for applications in sensors or energy storage devices.

### Building SA/TiO_2_ and SA/PPy/TiO_2_ model molecules

**Fig. 6:**
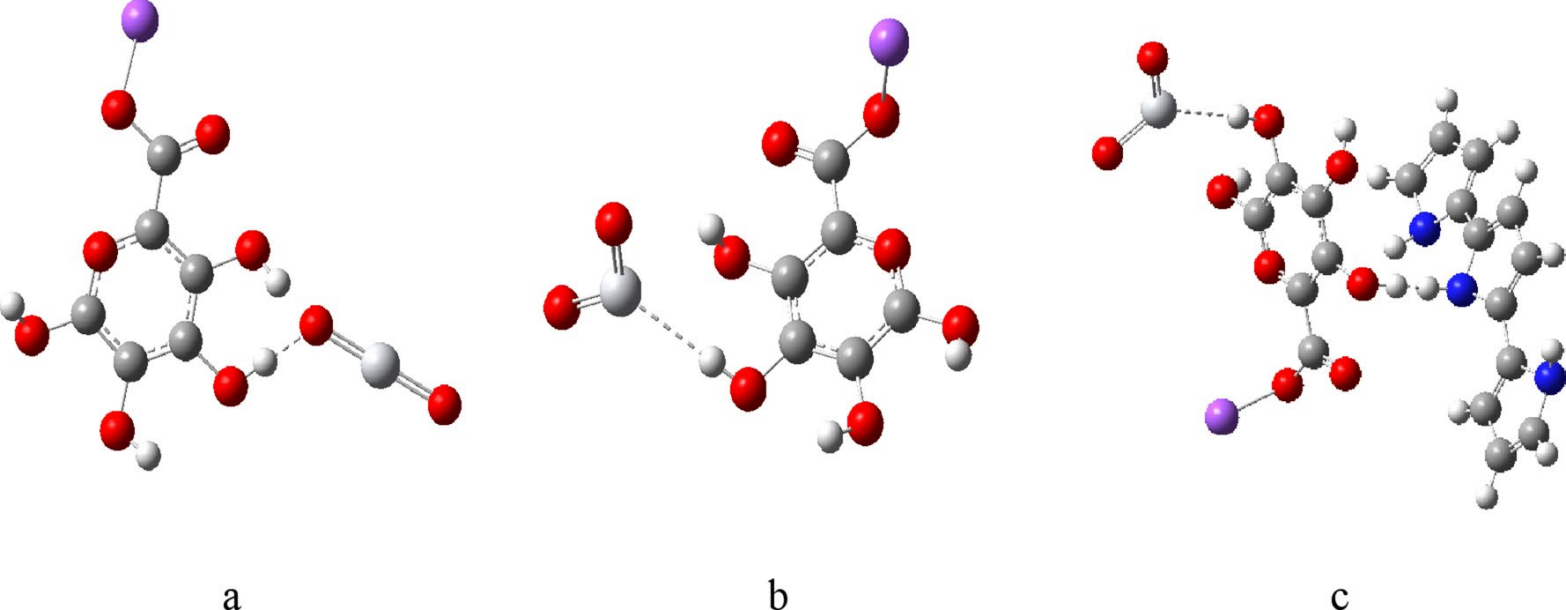
Proposed structures: (**a**) SA (H2) with TiO_2_ (O), (**b**) SA (H2) with TiO_2_ (Ti), (**c**) SA (H1, H3) and PPy (H1, M) with TiO_2_ (H3, Ti).

Moreover, two molecules were created for the interactions between SA and TiO_2_, one focusing on the bonding via oxygen (O) and the other via titanium (Ti) as depicted in Fig. 6 (a-b). Additionally, a molecule that includes all three materials together was constructed after determining the minimum binding energy between SA and PPy as depicted in Fig. 6 (c). By systematically analyzing all possible bonding configurations, deeper insights can be gained for the reactivity and interaction dynamics among sodium alginate, polypyrrole, and titanium dioxide, ultimately enhancing our understanding of their combined properties and potential applications.

Table 3 shows that the interaction of SA (H2) with TiO_2_ via (O) results in the highest TDM of 11.3730 Debye, indicating significant changes in polarity based on molecular arrangements. Additionally, the interaction of SA (H2) with TiO_2_ results in a band gap energy of 2.4397 eV, demonstrating that the integration of these materials can modulate electronic properties effectively. Overall, these values highlight the importance of molecular structure and interactions in determining the electronic behavior of these composites.

**Table 3:** Calculated TDM in (Debye), ΔE in (eV) for the studied SA/TiO_2_ and SA/PPy/TiO_2_ composite structures.

Structure	TDM (Debye)	ΔE (eV)
SA (H2) with TiO_2_ (O)	11.3730	2.4397
SA (H2) with TiO_2_ (Ti)	5.1078	3.3660
SA and PPy with TiO_2_	5.1096	1.6367

**Fig. 7:**
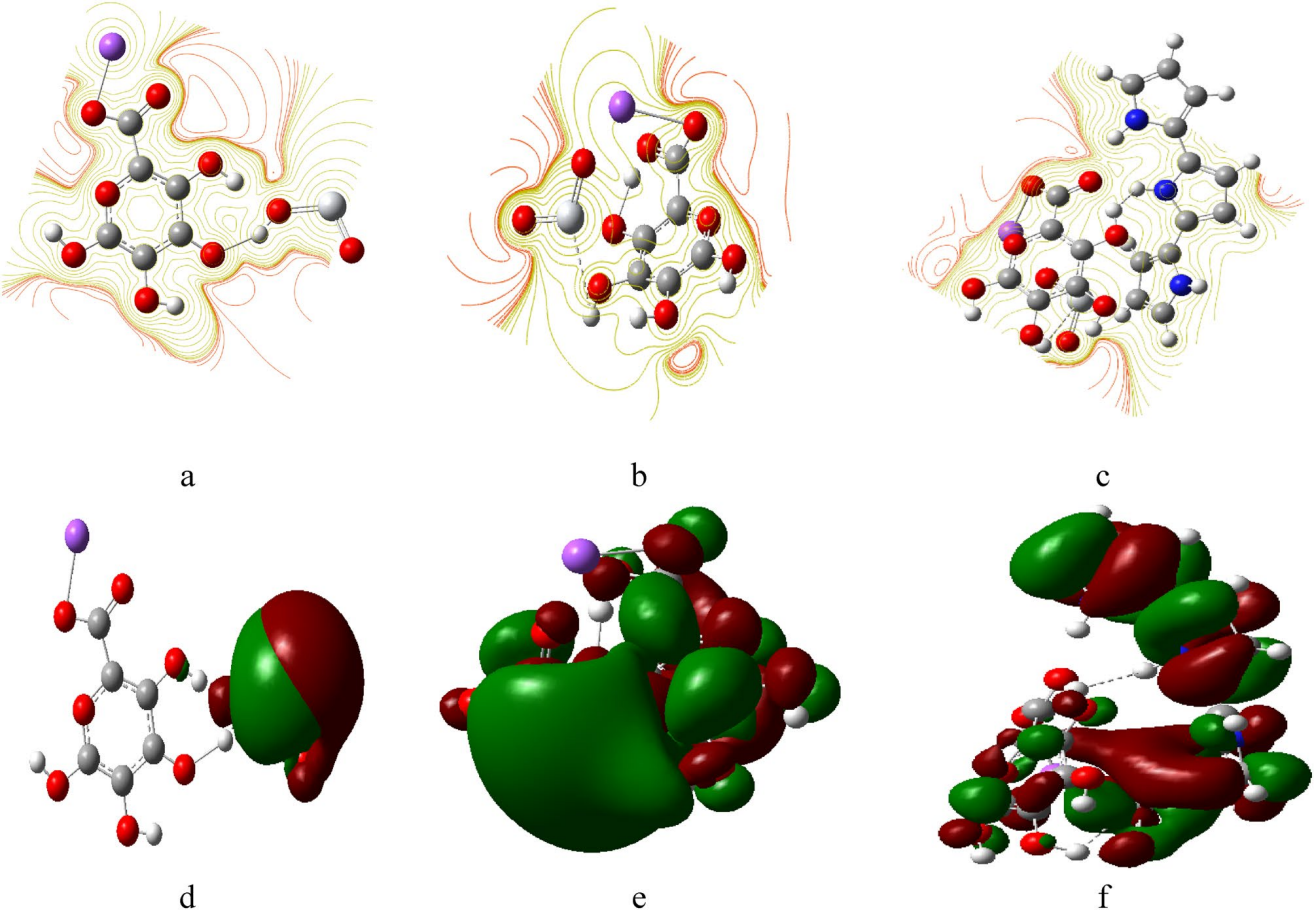
MESP maps for (**a**) SA (H2) with TiO_2_ (O), (**b**) SA (H2) with TiO_2_ (Ti), (**c**) SA (H1,H3) and PPy (H1,M) with TiO_2_ (H3,Ti), calculated HOMO/LUMO energy gaps for (**d**) SA (H2) with TiO_2_ (O), (**e**) SA (H2) with TiO_2_ (Ti), (**f**) SA (H1,H3) and PPy (H1,M) with TiO_2_ (H3,Ti).

Figure 7 shows the MESP and calculated HOMO/LUMO band gap energies for SA (H2) with TiO_2_ (O) (Fig. 7: a), SA (H2) with TiO_2_ (Ti) (Fig. 7: b), and SA (H1, H3) and PPy (H1, M) with TiO_2_ (H3, Ti) (Fig. 7: c) model structures. The presence of TiO₂ in the composite further showcases how these interactions can enhance or modify the electronic behavior, indicating potential pathways for improving material performance in different applications. Overall, the HOMO/LUMO energy gap analysis provides a comprehensive understanding of the electronic landscape of these materials, emphasizing the importance of molecular structure and interactions in determining their chemical properties.

### Calculated reactivity descriptors

The following formulas were used to derive global reactivity descriptors, such as Ionization potential (I) (eV), Electronic affinity (A) (eV), Electronic chemical potential (µ) (eV), Chemical hardness (η) (eV), Absolute softness (S) (eV^−1^), and Electrophilicity index (ω) (eV)^36,53^:

1 $$I=-{E}_{HOMO}$$

2 $$\:A=-{E}_{LUMO}$$

3 $$\:\mu\:=-\frac{I\:+A}{2}$$

4 $$\:\eta\:=\frac{I-A}{2}$$

5 $$\:S=\frac{1}{\eta\:}$$

6 $$\:\omega\:=\frac{{\mu\:}^{2}}{2\eta\:}$$

Table 4 shows the various electronic characteristics for SA, PPy, and TiO₂ as well as their different building molecules. The data presented provides an in-depth investigation of these characteristics.

The ionization potential (I) indicates how much energy was required to remove an electron from a molecule. At 3.011 eV, SA has the lowest value. Given that SA needs less energy to lose an electron than PPy and TiO₂, this suggests SA is more reactive. On the other hand, TiO₂, which is characteristic of transition metal oxides, shows the highest ionization potential at 6.501 eV, suggesting greater stability and resistance to electron loss. The electronic affinity (A), which measures a molecule’s tendency for gaining electrons, TiO₂ shows a value of 3.006 eV, demonstrating its strong electron-attracting abilities. This characteristic is essential when considering TiO₂ reactivity. In contrast, PPy exhibits a relatively low electronic affinity at 0.063 eV, indicating a reduced tendency for accepting electrons, which aligns with its stable nature as a conducting polymer. This property is significant in its function as a p-type organic semiconductor, which are materials that primarily transport positive charge carriers (holes). While electron affinity is a fundamental property of atoms and molecules, factors like nuclear charge and atomic size influence its value^54^.

The electronic chemical potential (µ) represents the molecule’s overall tendency to either gain or lose electrons. It is computed by averaging the ionization potential and electronic affinity. Values are negative for all structures, with TiO₂ having the lowest at −4.754 eV. This implies that TiO₂ has a lower electron loss rate than the others, consequently enhancing its stability. The tendency for electron loss is also indicated by the electrical potential values for SA and PPy; SA is more advantageous for electron donation due to its less negative value. A species’ resistance to changes in electron density is measured by chemical hardness (η), and PPy has the highest value at 2.191 eV. Under electron perturbations, this suggests that PPy is less reactive and more stable. SA, on the other hand, has less hardness, which suggests that it is more reactive in responding to changes in its electronic configuration. In terms of absolute softness (S), which measures a molecule’s ease of polarization. SA has a higher softness value of 1.414 eV^− 1^ compared to PPy’s 0.456 eV^− 1^. This suggests that SA is more easily polarized, which could enhance its interactions with other molecules or surfaces, making it potentially useful material in various applications. The ability of a molecule to take electrons is indicated by its electrophilicity index (ω), and TiO₂ has the highest index at 6.464 eV. According to this, TiO₂ is a strong electrophile and so highly reactive during electron transfer processes. In contrast, PPy is less reactive in this sense, as evidenced by its lower value of 1.159 eV.

The study of these electronic properties provides an in-depth understanding of how these composites’ electronic behavior and molecular structure affect their stability and reactivity.

**Table 4:** DFT:B3LYP/6-31G (d, p) calculated global reactivity descriptors: Ionization potential (I) (eV), Electronic affinity (A) (eV), Electronic chemical potential (µ) (eV), Chemical hardness (η) (eV), Absolute softness (S) (eV^− 1^) and Electrophilicity index (ω) (eV) for the studied structures.

Structure	I (eV)	A (eV)	µ (eV)	η (eV)	S (eV^− 1^)	ω (eV)
SA	3.011	1.597	−2.304	0.707	1.414	3.753
PPy	4.444	0.063	−2.254	2.191	0.456	1.159
TiO_2_	6.501	3.006	−4.754	1.748	0.572	6.464
SA (H1) with PPy (T)	3.458	1.751	−2.604	0.854	1.171	3.972
SA (H2) with PPy (T)	3.444	1.703	−2.574	0.870	1.149	3.806
SA (H3) with PPy (T)	3.643	1.765	−2.704	0.939	1.065	3.892
SA (H4) with PPy (T)	3.257	0.921	−2.089	1.168	0.856	1.867
SA (H1) with PPy (M)	3.325	1.654	−2.489	0.835	1.197	3.710
SA (H2) with PPy (M)	3.256	0.921	−2.089	1.168	0.856	1.868
SA (H3) with PPy (M)	3.532	1.728	−2.630	0.902	1.109	3.833
SA (H4) with PPy (M)	3.257	0.920	−2.088	1.168	0.856	1.867
SA (H2) with TiO_2_ (O)	4.539	2.099	−3.319	1.220	0.820	4.514
SA (H2) with TiO_2_ (Ti)	4.802	1.355	−3.079	1.723	0.580	2.750
SA and PPy with TiO_2_	4.508	1.841	−3.175	1.334	0.750	3.778

**Fig. 8:**
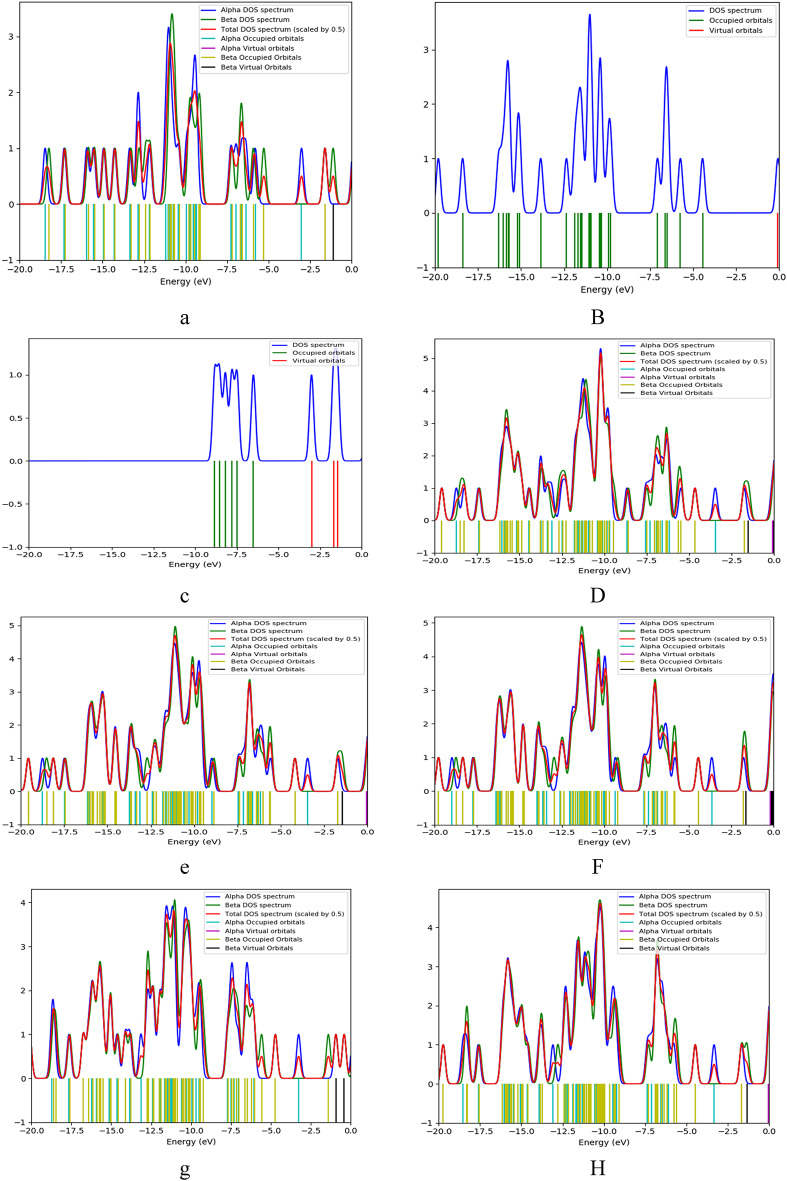

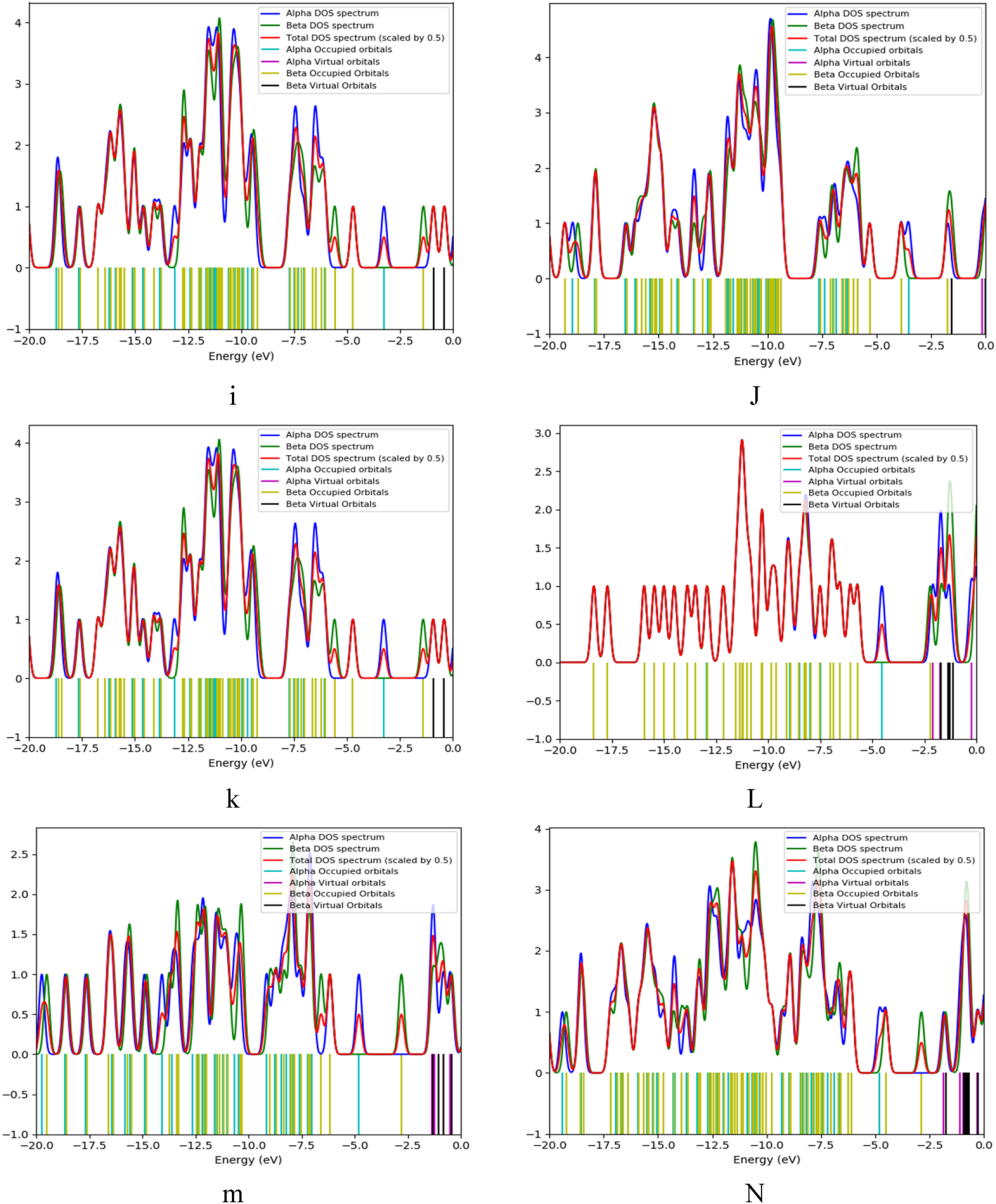
presents the density of states (DOS) providing a detailed understanding of the electronic structure of the studied model structures for SA, PPy, and TiO₂. The DOS graphs illustrate how the energy levels of the electrons are distributed within each structure.

Figure 8. The density of states (DOS) for studied structures for (**a**) SA, (**b**) PPy consists of 3 units, (**c**) TiO_2_, (**d**) PPy (T) with SA (H1), (**e**) PPy (T) with SA (H2), (**f**) PPy (T) with SA (H3), (**g**) PPy (T) with SA (H4), (**h**) PPy (M) with SA (H1), (**i**) PPy (M) with SA (H2), (**j**) PPy (M) with SA (H3), (**k**) PPy (M) with SA (H4), (**l**) SA (H2) with TiO_2_ (O), (**m**) SA (H2) with TiO_2_ (Ti), (**n**) SA (H1,H3) and PPy (H1,M) with TiO_2_ (H3,Ti).

Comparing the DOS of SA/TiO₂ (Fig. 8: l-m) blend with the SA/PPy/TiO₂ (Fig. 8: n) ternary composite shows that the addition of PPy leads to a significant reduction in the HOMO–LUMO band gap energy. This is because PPy (Fig. 8: b) adds new π-derived states above the valence band maximum and π* states below the conduction band minimum of the SA/TiO₂. The pi bonds forming gap states in the middle of the band gap, lower the activation barrier associated with the excitation of electrons, increasing charge carriers. Overall, the DOS analysis provides crucial insights into the electronic properties of the studied model structures, illustrating how molecular interactions and configurations can significantly affect their reactivity and stability. This understanding is essential for guiding the development of novel materials for various applications, such as sensors and electronic devices.

**Fig. 9:**
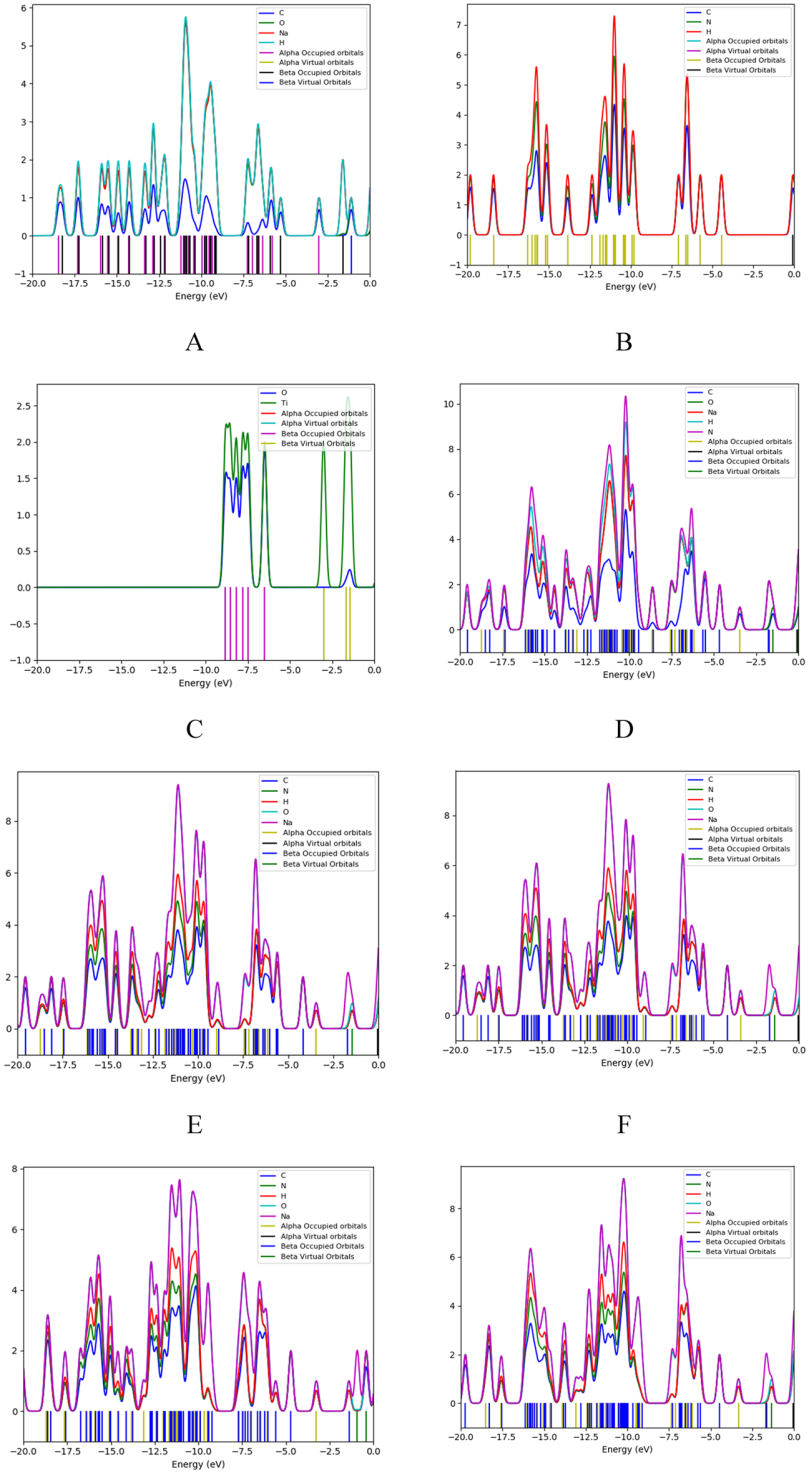

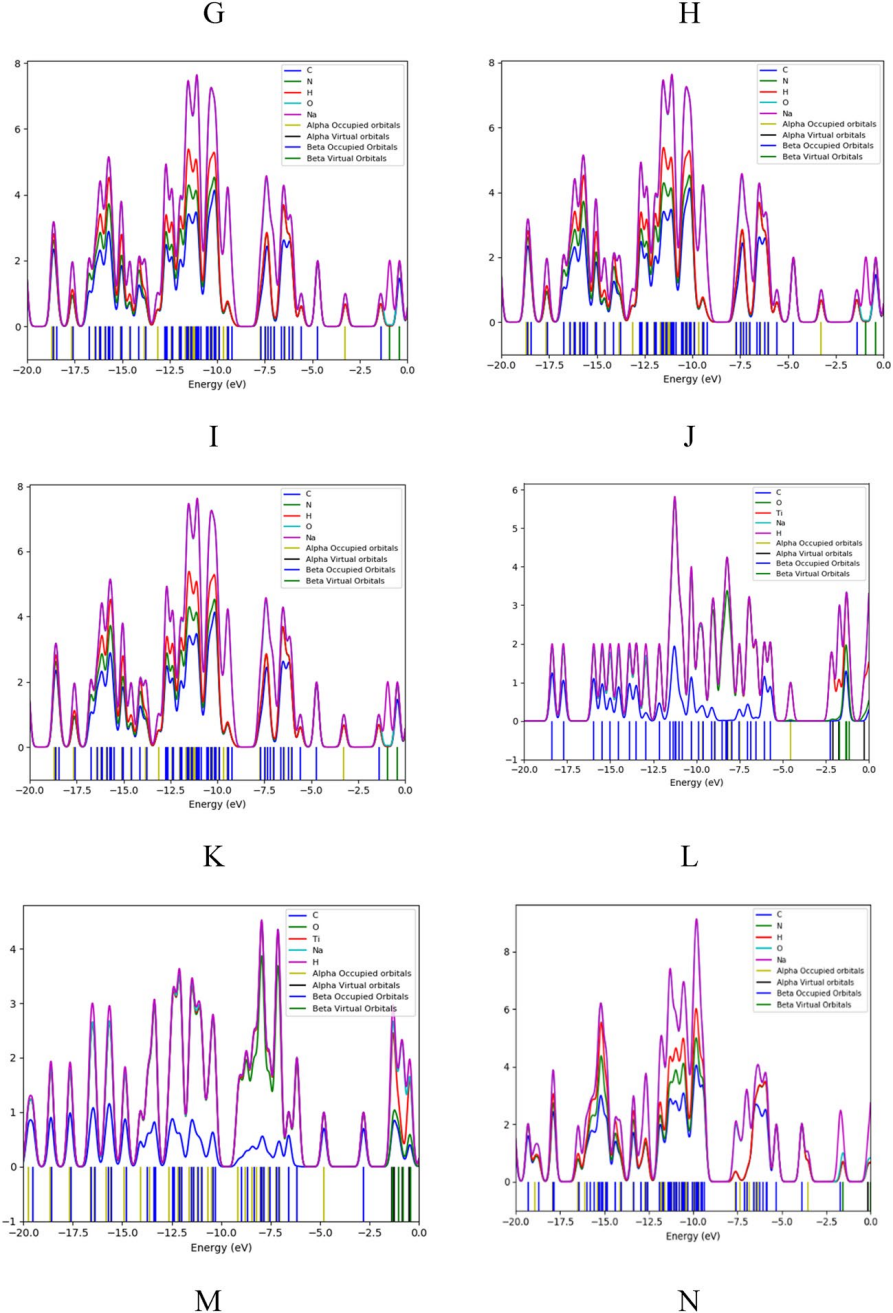
The projected density of states (PDOS) for studied structures for (**a**) SA, (**b**) PPy consists of 3 units, (**c**) TiO_2_, (**d**) PPy (T) with SA (H1), (**e**) PPy (T) with SA (H2), (**f**) PPy (T) with SA (H3), (**g**) PPy (T) with SA (H4), (**h**) PPy (M) with SA (H1), (**i**) PPy (M) with SA (H2), (**j**) PPy (M) with SA (H3), (**k**) PPy (M) with SA (H4), (**l)** SA (H2) with TiO_2_ (O), (**m**) SA (H2) with TiO_2_ (Ti), (**n**) SA (H1,H3) and PPy (H1,M) with TiO_2_ (H3,Ti).

**Fig. 10:**
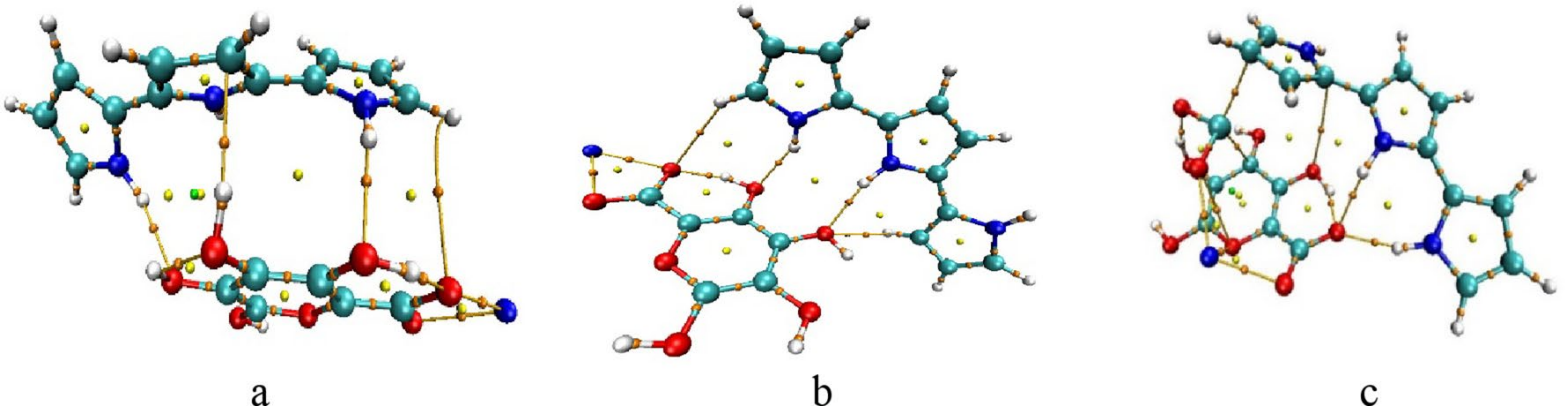
The Quantum Theory of Atoms in Molecules QTAIM for studied structures for (**a**) SA (H1) with PPy (T), (**b**) SA (H3) with PPy (M), (**c**) SA (H1,H3) and PPy (H1,M) with TiO_2_ (H3,Ti).

Figure 9 illustrates the Projected Density of States (PDOS) for various molecule structures arrangements involving PPy and SA. Each curve represents a different configuration, allowing for an analysis of how the electronic states are distributed among the components^55^. The PDOS analysis for SA/TiO₂ (Fig. 9: l-m) and SA/PPy/TiO₂ (Fig. 9: n) identify the orbital constituents responsible for the emergence of these new states. The valence band of SA/TiO₂ is primarily composed of O 2p orbitals from alginate and Ti 3p from TiO₂, while the conduction band comprises principally of Ti 3d. The introduction of PPy (Fig. 10: b) adds a N 2p–π and a π* band which overlaps with Ti 3 d, forming a new valence band^56^.

Figure 10 represents the Quantum Theory of Atoms in Molecules (QTAIM) analysis, providing deeper insights into bonding characteristics and electron density distribution within the studied structures. QTAIM is a powerful theoretical framework that allows for the examination of the electronic structure of molecules in terms of atomic contributions. It helps in visualizing how electron density is distributed among different atoms, offering insights into the nature of chemical bonding. In the provided Figures, QTAIM can reveal critical information about the interactions between PPy, SA, and TiO₂^57^.

Electron density at bond critical points (BCPs) between interacting atoms are essential for determining the strength of bonding interactions. Higher electron density values at these critical points generally indicate stronger electronic charge densities, corresponding to more stable and covalent interaction. Moreover, if the Laplacian charge density (∇2ρ(r)) is less than 0 and the total electron energy density (H(r)) is less than 0, it suggests a covalent (shared) interaction. However, when both ∇2ρ(r) and H(r) are greater than 0, it points to non-covalent (closed shell) interactions, such as weak hydrogen bonds, van der Waals forces, and electrostatic interactions. QTAIM analysis reveals the nature of non-covalent interactions within the studied structures. The analysis shows that the structure SA (H1) with PPy (T) (Fig. 10: a) is more stable than the structure SA (H3) with PPy (M) (Fig. 10: b). This is due to the non-covalent interactions, which are more prevalent in the former structure, such as electrostatic interactions and hydrogen bonding. These non-covalent interactions enable sodium alginate to stabilize the composite.

### Verification of the model

**Fig. 11:**
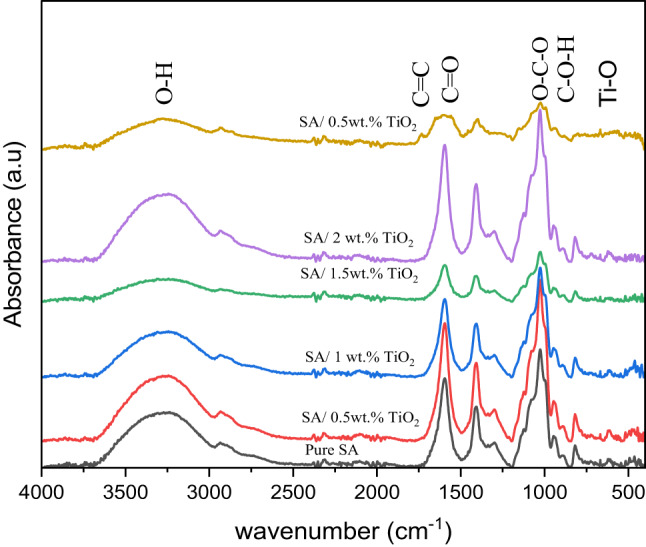
FTIR spectra for pure SA and SA/TiO₂ composite films with different concentrations of TiO₂.

FTIR spectroscopy was employed to investigate the structural features and interfacial interactions between SA and TiO₂. Figure 11 presents the FTIR absorption spectra of pure SA and SA/TiO₂ composite films within the spectral range of 4000–400 cm⁻¹. The observed shifts and changes in the spectral features confirm the presence of interfacial interactions between the organic (SA) and inorganic (TiO₂) components in the prepared systems^58^. The characteristic absorption bands observed in the spectra (Fig. 11) are consistent with those reported in the literature^59,60^. To gain further insight, the experimental FTIR data were compared with the scaled vibrational frequencies obtained from computed IR spectra. Table 5 summarizes the comparison between the calculated and experimental vibrational frequencies. The O-H stretching vibration, calculated in the range of 3656–3626 cm⁻¹, corresponds to the broad FTIR absorption band observed at 3290 cm⁻¹. The C = C stretching mode, predicted at 1671 cm⁻¹, aligns with the FTIR peak at 1739 cm⁻¹. Similarly, the C = O stretching vibration, observed experimentally at 1600 cm⁻¹, is assigned to the computed range of 1527–1506 cm⁻¹. The O–C–O stretching, detected at 1027 cm⁻¹ in the FTIR spectrum, is matched by the computed band at 1139 cm⁻¹. In the lower wavenumber region (900–400 cm⁻¹), Ti–O stretching modes were identified, corresponding to the calculated range of 1035–696 cm⁻¹. Additionally, the C–O–H bending mode, observed in the experimental spectrum at approximately 943 cm⁻¹, aligns with the calculated range of 979–887 cm⁻¹. The good agreement between the experimental and theoretical vibrational frequencies confirms the reliability and accuracy of the applied computational method in predicting the molecular structure and interactions within the SA/TiO₂ system^61^.

Overall, the FTIR findings supported by theoretical calculations demonstrate that strong interfacial interactions exist in the SA/TiO₂ composite, which are essential for tailoring the material’s physical properties, and could play a pivotal role in determining its performance in applications such as sensing, drug delivery, or biodegradable packaging.

**Table 5:** Computed and scaled computed IR compared with FTIR band vibrational frequencies.

Computed IR	Scaled computed IR	FTIR	Assignment
3805 ~ 3774	3656 ~ 3626	3290	O-H stretching
1739	1671	1739	C = C
1590 ~ 1568	1527 ~ 1506	1600	C = O
1186	1139	1034	O-C-O
1078 ~ 725	1035 ~ 696	900 ~ 400	Ti-O stretching
1019 ~ 924	979 ~ 887	943	C-O-H

**Fig. 12:**
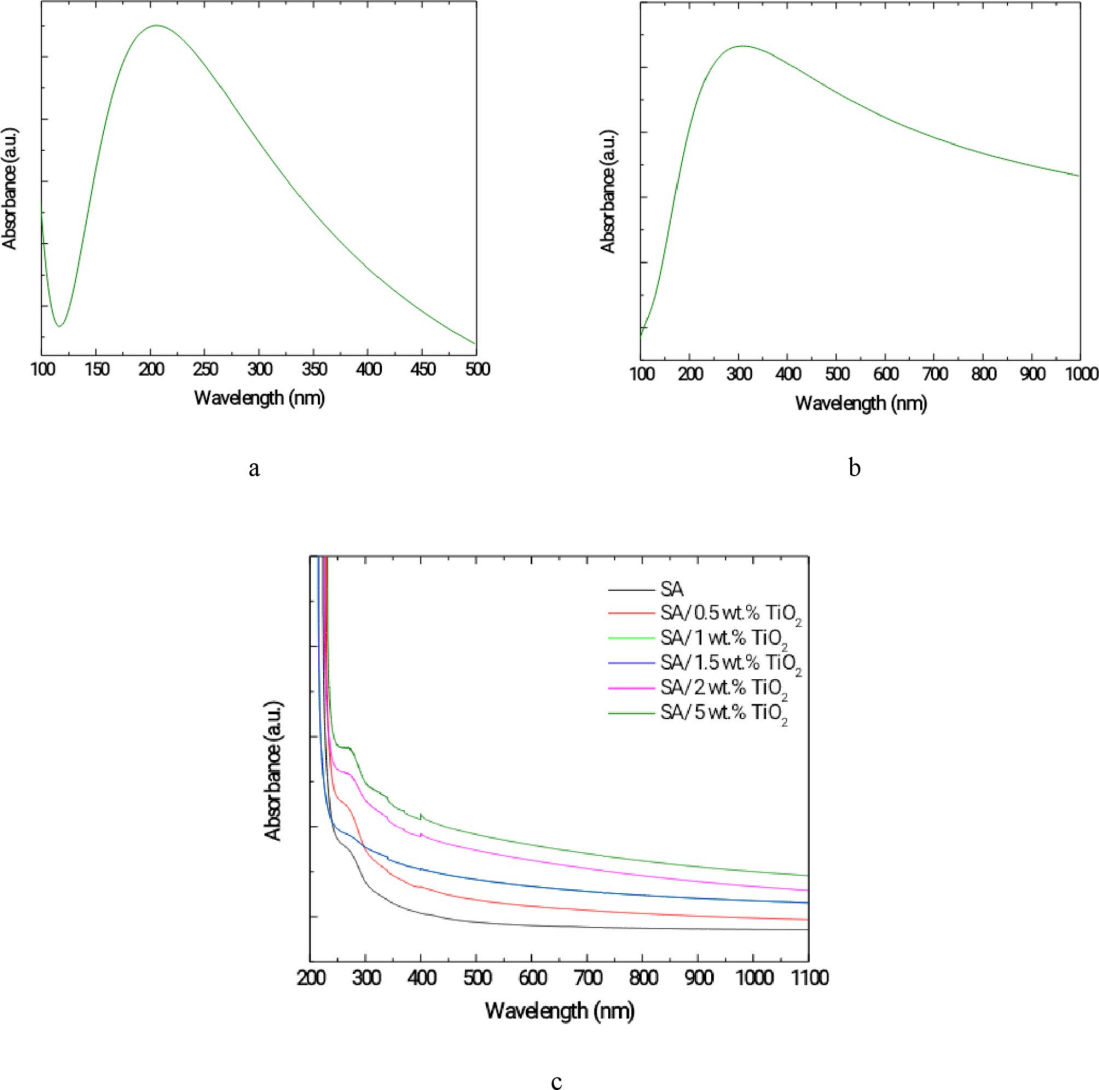
UV-Vis spectra for (**a**) TD-SCF and DFT/B3LYP/6-31G calculated spectrum of sodium alginate (SA), (**b**) TD-SCF and DFT/B3LYP/6-31G calculated spectrum of SA/TiO₂ complex, and (**c**) the experimental spectra of pure SA and SA/TiO₂ composite films with varying TiO₂ concentrations.

Figure 12 shows the comparison between the calculated UV-Vis absorption spectra of pure sodium alginate (SA) (Fig. 12-a) and its complex with TiO₂ (Fig. 12-b), obtained using the TD-SCF and DFT/B3LYP/6-31G method, along with the experimental spectra of SA/TiO₂ films with varying TiO₂ concentrations (Fig. 12-c). The theoretical spectra successfully reproduce the main absorption peaks observed experimentally, with only minor wavelength shifts. Specifically, the calculated maximum absorption for pure SA is 205 nm, compared to an experimental value of 260 nm, while for the SA/TiO₂ complex, is 308 nm, closely matching the experimental value of approximately 273 nm. These small deviations are commonly attributed to the neglect of explicit solvent effects and limitations of the applied basis set. The calculated spectrum of pure SA exhibits a prominent peak around 190–210 nm, attributed to π→π* transitions, which shifts to higher wavelengths upon TiO₂ incorporation, indicating significant electronic interactions and potential charge transfer. Experimentally, a noticeable increase in absorption intensity and a slight redshift are observed with increasing TiO₂ content, suggesting enhanced interfacial interaction and improved charge delocalization within the polymer matrix. The close agreement between calculated and experimental values confirms the reliability of the computational model in describing the electronic transitions of the studied systems and highlights the role of TiO₂ in tuning the optical properties of the SA matrix through efficient charge transfer mechanisms.

It was reported that, polypyrrole combined with TiO₂ produces better electronic performance because it decreases the bandgap and increases visible light absorption, which leads to better charge separation at the heterojunction interface^62^. The use of this conductive polymer–semiconductor combination in alginate hydrogels and aerogels results in mechanical stability and better dispersion and recyclability through the alginate framework, which keeps the semiconductor materials at their original band structure^63^. The strong interfacial interactions that occur between PPy and TiO₂ nanoparticles through hydrogen bonding create efficient electron transport pathways, which enhance the photocatalytic performance and overall system efficiency^64^. Moreover, out research results demonstrate that polypyrrole integration into the TiO₂ framework leads to better electronic performance through bandgap reduction and improved charge separation at the heterojunction interface. The observed results support the existing synergistic effects found in the ternary composite system because the alginate framework provides stability while the hydrogen-bonded interactions between PPy and TiO₂ create efficient electron transport pathways for higher system performance.

Another validation of the results will be conducted throughout dispersion analyses as in the following section.

### Dispersion analyses

Further validation for the studied model is performed with dispersion analyses. The SA/PPy/TiO_2_ structure was performed with B3LYP-D3 using Grimme’s third-generation dispersion correction, D3, to the B3LYP calculation GD3^65^. Table 6 presented the calculated TDM in (Debye), ΔE in (eV) for the studied SA/PPy/TiO_2_ with and without dispersion analyses. The results show comparable TDM values between the ones calculated with and without dispersion analysis, while the HOMO/LUMO band gap energy calculated with dispersion analysis decreased compared to its value without dispersion analysis.

**Table 6:** Calculated TDM in (Debye), ΔE in (eV) for the studied SA/PPy/TiO_2_ with and without dispersion analyses.

Structure	TDM (Debye)	ΔE (eV)
Without Dispersion	5.1096	1.6367
With Dispersion	6.4084	0.7717

The D3 correction is an empirical correction that is added to the total energy calculated by the DFT method. It is a computationally inexpensive add-on that significantly improves the accuracy of the results for systems where dispersion plays a role. It provides a better balance between computational cost and accuracy compared to more computationally demanding methods like Coupled Cluster theory, which are often considered the “gold standard” but are not feasible for larger systems as reported earlier^65^. It was reported that, this correction provides significant accuracy improvements at a low computational cost.

Correlating the above results, one can summarize the novelty of this study in some points. The research combines theoretical calculations (DFT) with experimental data (FTIR spectra) to create a more comprehensive and validated understanding of the material’s properties. This explores the synergistic effects of combining SA, PPy, and TiO₂, showcasing the potential for this nanocomposite in electronics and biomedical applications. The composite is found to have a significantly reduced energy gap and dipole moment, which are indicators of improved charge transfer and electronic reactivity. The research goes beyond basic analysis by using advanced techniques like Density of States (DOS) and Quantum Theory of Atoms in Molecules (QTAIM) to provide a deeper understanding of the molecular interactions. Finally, the study specifically identifies the role of PPy in lowering the HOMO-LUMO gap, which leads to a lower energy barrier for electron excitation and an increase in charge carriers, ultimately improving the composite’s electronic performance.

## Conclusion

DFT/B3LYP with the 6-31G(d, p) basis set was employed to elucidate the physical parameters of a sodium alginate/polypyrrole/titanium dioxide composite. Calculations of the Total Dipole Moment (TDM) and HOMO/LUMO energy gaps provided insight into the electronic structure of the model. Molecular electrostatic potential (MESP) analysis identified the most negative and positive surface areas, allowing for the prediction of hydrogen bonding sites that contribute to structural stability.

Regarding the studied individual components displayed a strategic balance of electronic properties such that:

Sodium alginate (SA) was identified as the most reactive material, exhibiting an ionization potential (I) of 3.011 eV.

Titanium dioxide (TiO_2_) demonstrated the highest stability at 6.501 eV, reinforced by a reduced tendency to lose electrons and a high electron affinity (A) of 3.006 eV.

Polypyrrole (PPy) showed its characteristic nature as a conducting polymer with a low affinity of 0.063 eV.

The SA/PPy/TiO_2_ composite merges these distinct characteristics, achieving a balance between reactivity and stability. The composite material shows potential for energy storage and environmental remediation based on its properties but requires additional testing to verify these applications.

The computational model was verified through the close alignment of computed IR frequencies with experimental FTIR data. The small differences between calculated results and actual measurements for the SA/TiO_2_ system ultraviolet spectra originated from the model’s basic design which did not include solvent effects. The model validation through dispersion analysis showed that TDM values remained constant while the HOMO/LUMO band gap energy decreased which produced a more precise description of the electronic characteristics in the system.

QTAIM analysis confirmed that structural stability is driven by non-covalent interactions. The SA (H1) with PPy (T) configuration showed better stability than the SA (H3) with PPy (M) alternative because it had more electrostatic and hydrogen bonding, which helped the SA matrix stabilize the composite.

The study establishes a strong theoretical-experimental correlation through its use of the B3LYP functional for the SA/TiO₂ system although the work demonstrates gas-phase approximation and ternary composite experimental characterization requirements as limitations which have to be addressed through future material optimization procedures.

The computational modeling results together with dispersion analysis results and experimental FTIR and UV-Vis spectrum results demonstrate that this method successfully validates the molecular interaction study of SA/PPy/TiO_2_ nanocomposite. The material qualifies as a theoretical candidate because it’s stabilizing non-covalent interactions and electronic profiles support its use in advanced energy technologies and environmental cleanup methods and biomedical material research.

## Data Availability

The data that support the findings of this study are available from the corresponding author upon reasonable request.
